# Association Between Statewide COVID-19 Lottery Announcements and Vaccinations

**DOI:** 10.1001/jamahealthforum.2021.3117

**Published:** 2021-10-15

**Authors:** Dhaval Dave, Andrew I. Friedson, Benjamin Hansen, Joseph J. Sabia

**Affiliations:** 1 Department of Economics, Bentley University, Waltham, Massachusetts; 2 Department of Economics, College of Liberal Arts and Sciences, University of Colorado Denver, Denver; 3 Department of Economics, College of Arts and Sciences, University of Oregon, Eugene; 4 Center for Health Economics & Policy Studies, College of Arts and Letters, San Diego State University, San Diego, California

## Abstract

This case-control study assesses if announcements of cash drawings in 19 states were associated with increased vaccine uptake by comparing vaccination trends in states that announced drawings with states that did not using a difference-in-differences framework.

## Introduction

In the United States, the COVID-19 vaccination rate slowed from a peak of 3.6 million vaccinations per day during the week of April 5, 2021, to fewer than 2 million vaccinations per day by the week of May 3, 2021. To boost vaccine uptake, 19 states announced large cash lotteries by July 1, 2021, that were tied to COVID-19 vaccination.

For instance, on May 12, 2021, Ohio announced Vax-a-Million, a set of weekly $1 million drawings to be held over 5 weeks for Ohio residents 18 years or older who had received at least 1 COVID-19 vaccine dose. One recent study failed to find an association between the Ohio drawings and increased vaccinations.^1^ In this case-control study, we assessed if announcements of cash drawings in 19 states were associated with increased vaccine uptake by comparing vaccination trends in states that announced drawings with states that did not using a difference-in-differences framework.

## Methods

We used daily state-level COVID-19 vaccination data from the Johns Hopkins University Vaccine Tracker between April 28 and July 1, 2021, a period during which vaccine appointments were widely and rapidly available.^2^ These data were combined with announcement dates from public news reports. We also collected COVID-19 case counts from *The New York Times* and restaurant and bar foot traffic from SafeGraph Inc.

We used a difference-in-differences framework for analysis, which compared daily reported COVID-19 vaccinations administered per 1000 population before and after the announcement in the 19 states that announced a lottery with those in states that never announced, while controlling for covariates. Details of the model can be found in eMethods in the Supplement. We also estimated event-study models, which interacted the difference-in-differences estimator with binary indicators for the number of days prior to or after the announcement. These estimates allowed us to assess how the association between announcements and vaccinations changed over time.

Analyses of secondary, deidentified data are exempt from institutional review board review, as determined by the University of Colorado. This study followed the Strengthening the Reporting of Observational Studies in Epidemiology (STROBE) reporting guidelines.

## Results

There were 37.2 million first doses of COVID-19 vaccine administered in the United States between April 28 and July 1, 2021, including 19.2 million in states that announced cash drawings. Ordinary least squares estimates of the difference-in-differences model are summarized in the Table. Estimates of the association between an announcement and vaccination rates were very small in magnitude and statistically indistinguishable from zero. For example, for first doses, the adjusted regression estimate was −0.06 (95% CI, −0.43 to 0.30) daily vaccinations per 1000 population.

**Table.** Changes in Daily State-Level COVID-19 Vaccinations per 1000 Population After Announcement of a Cash Lottery^a^

Dose	All states^b^	States with ≥14 d of postannouncement data and control states^b^
First dose^c^		
Difference-in-differences estimate of daily vaccinations per 1000 population (95% CI)^d^	−0.08 (−0.45 to 0.28)	−0.06 (−0.43 to 0.30)
Observations, No.	3312	3247
Preannouncement mean daily vaccinations per 1000 population	2.17	2.24
Second dose		
Difference-in-differences estimate of daily vaccinations per 1000 population (95% CI)^d^	−0.20 (−0.53 to 0.12)	−0.20 (−0.52 to 0.13)
Observations, No.	3315	3250
Preannouncement mean daily vaccinations per 1000 population	3.12	3.19

^a^ Weighted regression estimated using ordinary least squares where the dependent variable was state-by-day number of COVID-19 vaccinations per 1000 population and the cash-lottery announcement variable was set equal to 1 on the day of an official state government announcement that a cash drawing would be held, and for each day thereafter, but was set equal to 0 otherwise.

^b^ Control variables include 50 state dummies, 64 day dummies, foot traffic at restaurants and bars per 1000 state population 7 days prior, average new COVID-19 cases per 1000 population 7 days prior, percentage of state population covered by a local cash drawing, and an indicator for statewide noncash or in-kind lotteries.

^c^ Vaccination data on first doses for June 24, 25, and 26, 2021, in Oklahoma were omitted owing to unreliable provision of vaccination data.

^d^ 95% CIs are calculated using standard errors clustered on state.

This finding was reinforced by the event-time estimates plotted in the Figure.^3^ No statistically significant association was detected between a cash-drawing announcement and the number of vaccinations before or after the announcement date, a period that included announcements of lottery winners for most lottery states. This finding was evident using a conventional event-time model (Figure, A) and a novel technique that better accounts for staggered announcements (Figure, B).

**Figure.**
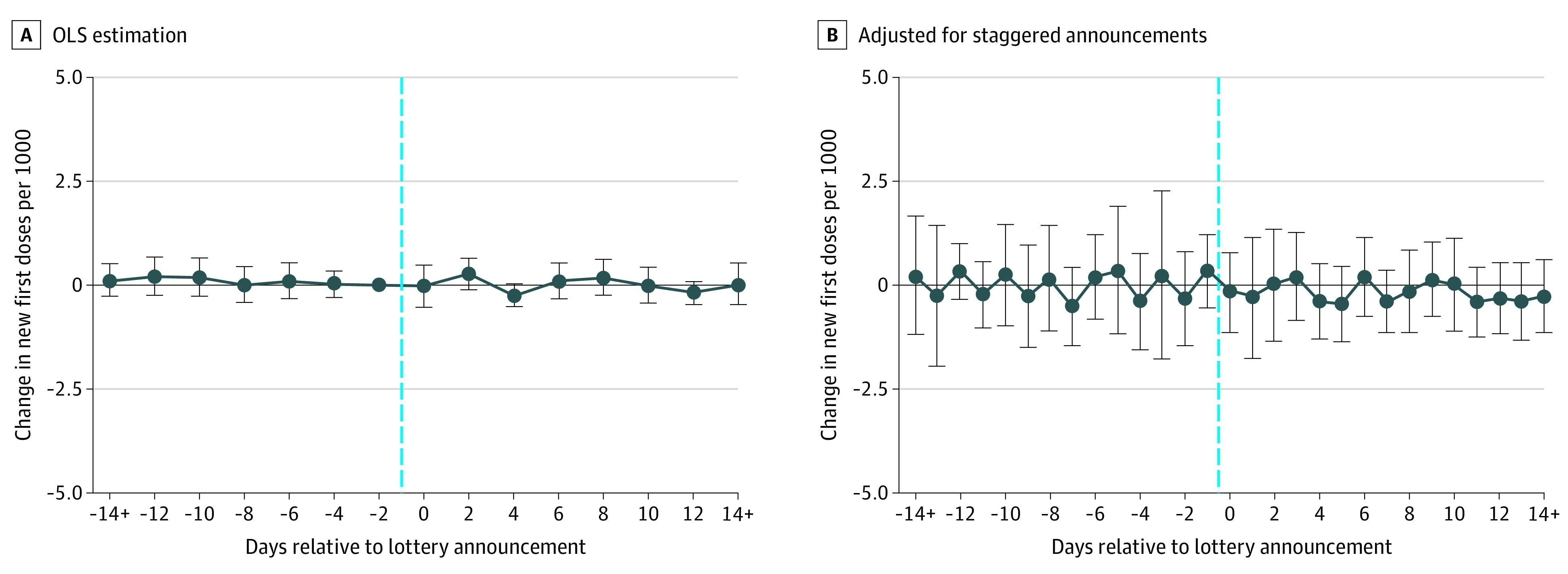
Event-Time Estimates for Announcement of a Cash Lottery Estimates with ordinary least squares (OLS) (A) and the method of Callaway and Sant’Anna^3^ using states that had not yet announced as counterfactuals (n = 3315) (B). Estimates are reported with vertical bars showing 95% CIs. The vertical dashed lines mark the day before the lottery was announced. Each estimate summarizes the association between the cash-drawing announcement and the daily vaccination rate at each point in time relative to the announcement. Ordinary least squares regressions include controls for 50 state indicators, 64 day indicators, foot traffic at restaurants and bars per 1000 state population 7 days prior, the share of the state population covered by a local cash vaccine lottery, an indicator for statewide noncash or in-kind lotteries, and average new COVID-19 cases per 1000 population 7 days prior. The omitted category was 2 days prior to the cash-drawing announcement.

## Discussion

With the upper bound of the 95% CI for the first-dose estimate of 0.30 daily vaccinations per 1000 population, we can rule out fairly small associations between lottery announcements and vaccinations. If the cash-drawing announcements had been associated with 70% of US adults receiving a first vaccine dose by July 4 (President Biden’s stated goal), we would have expected an estimate of approximately 1.22 daily vaccinations per 1000 population.

Results of this case-control study may reflect several factors. Lottery-style drawings may be less effective than incentives that pay with certainty. Another possibility is that drawings were not an informative vaccine promotional strategy and that more complete messaging on vaccination would have been far more effective.^4,5^ Also, individuals who are hesitant to receive COVID-19 vaccinations may be influenced by vaccine misinformation. This study is subject to limitations intrinsic to all analyses with nonexperimental data, and the potential for confounding factors remains. Moreover, these findings do not necessarily generalize to incentives for other vaccines.^6^

## Notes

Supplement. eMethods eReferences
