# Supplementary material for: Association Between Statewide COVID-19 Lottery Announcements and Vaccinations
Source: JAMA Health Forum. 2021 Oct 15;2(10):e213117. doi: 10.1001/jamahealthforum.2021.3117 (PMC8727031; doi:10.1001/jamahealthforum.2021.3117)
Supplement: Supplement. — eMethods eReferences [file jamahealthforum-e213117-s001.pdf]

Supplemental Online Content

Dave D, Friedson AI, Hansen B, Sabia JJ. Association between statewide COVID-19 lottery announcements and vaccinations. *JAMA Health Forum*. 2021;2(10):e213117. doi:10.1001/jamahealthforum.2021.3117

eMethods
eReferences

This supplemental material has been provided by the authors to give readers additional information about their work.

© 2021 Dave D et al. *JAMA Health Forum.*

Online Supplement

eMethods

We collect data from several sources. Our primary outcome variable is the number of COVID-19 vaccinations given each day in each state. We collected these data from public reports from the Centers from Disease Control and Prevention as reported by the Johns Hopkins University Vaccine Tracker. All of these data can be retrieved here: https://coronavirus.jhu.edu/about/how-to-use-our-data. We separately track the number of first doses administered (of any COVID-19 vaccine) and the number of second doses administered (for vaccines requiring a second dose). We dropped 3 observations (first doses for June 24, 25, and 26 in Oklahoma) due to data abnormalities (counts of given vaccinations were implausibly large).

Our primary explanatory variable is the date a state gubernatorial official announced its cash drawing. The criteria for inclusion is that the state must announce a cash drawing for those in the state who received a dose of COVID-19 vaccine, or announce that those who received a dose of the COVID-19 vaccine were entered into a cash drawing (the second definition includes states such as New York which gave those who were vaccinated lottery tickets).

We compiled our list using Hassan and Kannapell (2021) and National Governor's Association (2021) as primary sources, and then used Google-based web searches to validate the nature and announcement date of each cash drawing, eTable1 below details the dates of each announcement.

Announcement Dates

State	Announcement Date
Arkansas	May 25
Colorado	May 25
California	May 27
Delaware	May 25
Illinois	June 17
Kentucky*	May 10
Louisiana	June 17
Maine	June 16
Maryland	May 20
Massachusetts	June 15
Michigan	July 1
Nevada	June 17
New Mexico	June 1
New York	May 20
North Carolina	June 10
Ohio	May 12
Oregon	May 21
Washington	June 3
West Virginia	June 1

© 2021 Dave D et al. *JAMA Health Forum.*

*On May 10, Kentucky offered a coupon for a free lottery ticket ($225,000 maximum cash award to winner) for those ages 18+ who received a COVID-19 vaccine at over 180 Kroger and WalMart locations statewide. On June 4, Kentucky announced a "Shot at a Million" lottery for anyone in the state who had received a COVID-19 vaccine. Our findings are not sensitive to recoding the KY lottery announcement date to June 4.

We draw control variables from several sources. The first is daily state-level COVID-19 reported cases. These are publicly available through the *New York Times* and can be accessed here: https://github.com/nytimes/covid-19-data. For each state we use the seven-day moving average of COVID-19 cases per 1,000 population from 1 week prior as a control for the severity of that state's COVID-19 epidemic (which could influence individual attitudes towards COVID-19 vaccines).

Our second set of control variables come from the SafeGraph Point of Interest (POI) data series. These data are free to academic researchers but must be applied for here: https://www.safegraph.com/academics. The POI data counts cell phone pings at points of interest - specifically businesses, which can be classified by type using six-digit North American Industry Classification System (NAICS) industry codes. For each state date we aggregate up the number of pings at bars and at restaurants. We then use the number of pings per 1,000 population from 1 week prior as a control for general sentiment about infection risk and willingness to engage with others (which could influence individual attitudes towards COVID-19 vaccines).

Our third set of controls accounts for states having state-wide non-cash/in-kind drawings. For these we again use Hassan and Kannapell (2021) and National Governor's Association (2021) as primary sources, and then used Google-based web searches to validate the nature and announcement date of each non-cash drawing.

Our final set of controls accounts for states not having state-wide cash drawings, but having a substantial portion of the population covered by a local cash drawing. We again used Google-based web searches for each state, county and city sequentially to find cash drawings that meet our above criteria but at the local level. We then used U.S. Census Bureau (2020) estimates of state and county population to calculate the proportion of the state population that was influenced by a local drawing.

Detailed Description of Empirical Models

Our estimation strategy follows a difference-in-differences approach (see Dimick and Ryan 2014), which compares states with lotteries to those which did not before and after the announcements. The main advantage of this type of estimation is that it uses a comparison group so that any observed changes in outcomes in states with cash drawings are not interpreted in a vacuum. We estimate the following equation via ordinary least squares,

$$Y_{st} = \alpha + \beta_1 DD_{st} + \beta_2 X_{st} + \theta_s + \theta_t + \varepsilon_{st}$$

© 2021 Dave D et al. *JAMA Health Forum.*

where Y_{st} is the number of doses of COVID-19 vaccine per 1,000 population reported given in state *s* on date *t*. DD_{st} is the difference-in-differences estimator, a variable which takes on the value of one if state *s* announced a lottery-style cash drawing on or prior to date *t* and takes on the value of zero otherwise. X_{st} is a set of control variables for each state on each date; they include foot traffic at restaurants and bars per 1,000 state population seven days prior, average new COVID-19 cases per 1,000 population seven days prior, and percent of state population covered by a local cash drawing, and an indicator for statewide non-cash/in-kind lotteries. θ_s is a set of state fixed effects, that is a set of dummy variables for each state, which control for date invariant state characteristics (such as demographic makeup of the state). θ_t is a set of date fixed effects, that is a set of dummy variables for each day, which control for state invariant date characteristics (such as national policy recommendations from the Centers for Disease Control and Prevention).

The event-time version of this model is estimated via the following equation,

$$Y_{st} = \alpha + \sum_{i=k}^{K} \beta_i \left(Announce_s \times D_i\right) + \beta_2 X_{st} + \theta_s + \theta_t + \varepsilon_{st}$$

Where *i* indexes days relative to the announcement date, so *i=0* is the date of the announcement, *i=1* is the date after the announcement, *i=-1* is the date before the announcement and so forth. Y_{st} is again the number of doses of COVID-19 vaccine per 1,000 population reported given in state *s* on date *t*. $Announce_s$ is a variable which takes on the value one if the state ever announces a cash drawing, and zero otherwise. D_i is a variable which takes on a value of one if the observation is for date *i* relative to announcement and zero otherwise. All other variables are defined as above.

Figure 1 plots the estimates and 95% CIs for the β_i coefficients. In the period after announcement $(i > 0)$, these coefficients represent the estimated association between having announced a lottery and the outcome (the incident immunization rate per 1,000) at *i* days post announcement. Prior to the announcement $(i < 0)$, the coefficients represent the estimated association between announcing a lottery and the outcome at *i* days prior to announcement.

Any estimate that is statistically different from zero during this time period could be considered evidence that states with and without cash drawings had systematically different patterns in the outcome variable leading up to the announcement. This is known empirically as the existence of non-parallel trends in the pre-period. One of the underlying assumptions of valid difference-in-differences estimation is that there are parallel trends between "treated" states (in this case states that announced a cash drawing) and "control" states (in this case states that did not). Thus, the estimates where $(i < 0)$ serve as a test for violations of the parallel trends assumption.

Panel B of Figure 1 repeats estimation of the event-time model using methods developed by Callaway and Sant'Anna (2020) that adjust for bias in difference-in-differences models that may be present when the event being studied occurs at different times in different places and there are heterogeneous dynamic treatment effects (see Goodman-Bacon 2021), as could, in theory, be the case with state cash drawing announcements. Our approach uses counterfactuals for treatment

© 2021 Dave D et al. *JAMA Health Forum.*

states that never announced or had not yet announced a vaccination lottery ("not yet adopters"). The findings are not sensitive to restricting the counterfactual states to only states that never announced a vaccine lottery over the April 28th through July 1st period.

Detailed Description of Calculation in Discussion Section

In the Discussion Section we compare the upper bound of our 95% CI of 0.30 to a hypothetical value of 1.22 which would be the size of the estimate we would expect had the state cash drawings generated enough new vaccinations to reach the President's goal of 70% of the adult population with a first vaccine dose by July 4th. What follows is how we arrived at this number. The calculation is not meant to be an exact calculation, but a ballpark number to demonstrate the magnitude of impact needed and that our upper 95% CI was not close.

For the states that announced, on the day prior to the lottery announcement, the cumulative one-dose vaccination rate was 647.9 per 1,000 adults. That implies that 64.8% of the adult population in the treated states had at least one dose, on the day prior to the lottery announcement.

So the pre-announcement 1 dose vaccination rate was 64.8%, and with a mean adult population in these states of 6,420,582 this implies a total number of adults with at least 1 dose was 4,160,537.

The target amount is to have 70% with at least 1 dose, which with the same mean population would imply 4,494,407 people with at least 1 dose. This means that the policy would need to increase first-dose vaccinated adults on average by 333,870 for the average treated state over 33 days (from June 2nd till July 4th).

Our outcome is in terms of the daily incidence rate (per 1000 population). Thus, we divide the required increase in first-dose vaccinated adults (333,870) by the average treated state population, and convert to per 1,000.

(333,870 / 8,280,780)*1,000 = 40.32

This is the total increase in the incidence rate needed by July 4th.

Since our effect is the average effect on daily incidence, we divide 40.32 by the number of days (33 days between June 2nd and July 4th):

40.32 / 33 = 1.22

This is the effect size that would be required to close the gap.

© 2021 Dave D et al. *JAMA Health Forum.*

eReferences

Callaway, B. and Sant'Anna, P.H., 2020. Difference-in-differences with multiple time periods. J of Econometrics.

Dimick, J.B. and Ryan, A.M., 2014. Methods for evaluating changes in health care policy: the difference-in-differences approach. JAMA, 312(22), pp.2401-2402.

Goodman-Bacon, A., 2021. Difference-in-differences with variation in treatment timing. J of Econometrics.

Hassan, K., and Kannapell, A. July 3, 2021. These are the U.S. states trying lotteries to boost COVID vaccinations. *The New York Times.*

National Governors Association. July 30, 2021. COVID-19 Vaccine Incentives. Available at: https://www.nga.org/center/publications/covid-19-vaccine-incentives/

U.S. Census Bureau. 2020. Population and Housing Unit Estimates Tables. Available at: https://www.census.gov/programs-surveys/popest/data/tables.2019.html

© 2021 Dave D et al. *JAMA Health Forum.*
